# IgM-Enriched Immunoglobulin Attenuates Systemic Endotoxin Activity in Early Severe Sepsis: A Before-After Cohort Study

**DOI:** 10.1371/journal.pone.0160907

**Published:** 2016-08-09

**Authors:** Saskia Wand, Matthias Klages, Christin Kirbach, Joanna Warszawska, Patrick Meybohm, Kai Zacharowski, Alexander Koch

**Affiliations:** 1 Department of Anesthesia, Intensive Care Medicine and Pain Therapy, University Hospital Frankfurt, Frankfurt am Main, Germany; 2 Clinic for Anaesthesiology, University Hospital Goettingen, Goettingen, Germany; Medizinische Universitat Graz, AUSTRIA

## Abstract

**Introduction:**

Sepsis remains associated with a high mortality rate. Endotoxin has been shown to influence viscoelastic coagulation parameters, thus suggesting a link between endotoxin levels and the altered coagulation phenotype in septic patients. This study evaluated the effects of systemic polyspecific IgM-enriched immunoglobulin (IgM-IVIg) (Pentaglobin^®^ [Biotest, Dreieich, Germany]) on endotoxin activity (EA), inflammatory markers, viscoelastic and conventional coagulation parameters.

**Methods:**

Patients with severe sepsis were identified by daily screening in a tertiary, academic, surgical ICU. After the inclusion of 15 patients, the application of IgM-IVIg (5 mg/kg/d over three days) was integrated into the unit’s standard operation procedure (SOP) to treat patients with severe sepsis, thereby generating “control” and “IgM-IVIg” groups. EA assays, thrombelastometry (ROTEM^®^) and impedance aggregometry (Multiplate^®^) were performed on whole blood. Furthermore, routine laboratory parameters were determined according to unit’s standards.

**Results:**

Data from 26 patients were included. On day 1, EA was significantly decreased in the IgM-IVIg group following 6 and 12 hours of treatment (0.51 ±0.06 vs. 0.26 ±0.07, p<0.05 and 0.51 ±0.06 vs. 0.25 ±0.04, p<0.05) and differed significantly compared with the control group following 6 hours of treatment (0.26 ±0.07 vs. 0.43 ±0.07, p<0.05). The platelet count was significantly higher in the IgM-IVIg group following four days of IgM-IVIg treatment (200/nl ±43 vs. 87/nl ±20, p<0.05). The fibrinogen concentration was significantly lower in the control group on day 2 (311 mg/dl ±37 vs. 475 mg/dl ±47 (p = 0.015)) and day 4 (307 mg/dl ±35 vs. 420 mg/dl ±16 (p = 0.017)). No differences in thrombelastometric or aggregometric measurements, or inflammatory markers (interleukin-6 (IL-6), leukocyte, lipopolysaccharide binding protein (LBP)) were observed.

**Conclusion:**

Treatment with IgM-enriched immunoglobulin attenuates the EA levels in patients with severe sepsis and might have an effect on septic thrombocytopenia and fibrinogen depletion. Viscoelastic, aggregometric or inflammatory parameters were not influenced.

**Trial Registration:**

clinicaltrials.gov NCT02444871

## Introduction

Endotoxin (lipopolysaccharide (LPS)) is a cell wall component of gram-negative bacteria. Elevated LPS concentrations in the bloodstream trigger pathophysiological cascades of sepsis and septic shock [1, 2]. With systemic inflammation leading to hypoperfusion of the gastrointestinal tract, which is an immense reservoir of endotoxin, the presence of LPS in the bloodstream is not necessarily associated with gram-negative infections [3–5].

Physiologically, endotoxin is neutralized by crosslinking immunoglobulin class M (IgM), which facilitates phagocytosis and elimination. The human polyspecific immunoglobulin preparation, Pentaglobin^®^, is enriched in immunoglobulin class M (IgM) and thus seems capable of neutralizing bacterial endotoxins. This effect has been demonstrated in *ex vivo* experiments and a randomized controlled clinical trial [6, 7]. Though the effects of IgM-enriched immunoglobulins (IgM-IVIg) on endotoxin levels in patients with sepsis have been investigated using the Limulus Amebocyte Lysis test (LAL) [8]. A more recent method of endotoxin measurement, the EA assay (EAA), has not been used to evaluate the effects of IgM-IVIg on the endotoxin levels. EAA has been shown to be more precise and robust than the LAL test [9].

A dysbalance of the pro- and anticoagulation systems, which can lead to a disseminated intravascular coagulation, seems to be a major pathophysiology in septic patients [10, 11]. Coagulation markers, such as the international normalized ratio (INR), activated partial thromboplastin time (aPTT), platelet count and fibrinogen concentration, are altered during systemic inflammation and infection [12–14]. Furthermore, viscoelastic and aggregometric parameters are affected. Adamzik et al. demonstrated that parameters of a functional coagulation analysis using rotational thrombelastometry (ROTEM) could predict the 30-day mortality more accurately than standard scoring systems, such as the simplified acute physiology score or the sequential organ failure assessment (SOFA) [15]. The presence of endotoxin both *in vitro* and *in vivo* can modulate several ROTEM parameters to a more procoagulatory state, e.g. decreasing the clotting time (CT) [16, 17]. Furthermore, a correlation between the measured endotoxin activity (EA) levels and the functional coagulation parameters, e.g., CT and clot formation time (CFT), has been demonstrated in patients with systemic inflammatory response syndrome (SIRS) or sepsis [18].

In this before-after cohort study, we aimed to investigate the effects of IgM-IVIg (Pentaglobin^®^) therapy on EA in patients with severe sepsis or septic shock as a primary endpoint. Secondary endpoints focused on the possible effects of IgM-IVIg therapy on the functional coagulation parameters, as measured by ROTEM and multiple electrode aggregometry (MEA), and on the conventional coagulation parameters and the inflammatory markers, such as INR, aPTT, platelet count, fibrinogen concentration, LBP, Interleukin (IL)-6 levels and leukocyte counts.

## Materials and Methods

### Study Design and Patients

This single-center before-after cohort study was conducted in a 34-bed tertiary academic surgical ICU from January to June 2013 at the University Hospital Frankfurt am Main, Germany. The study complies with the declaration of Helsinki and was approved by the local Scientific and Ethics Review Board (Ethics Committee of the medical faculty of the Johann Wolfgang Goethe-University, Theodor-Stern-Kai 7, 60590 Frankfurt am Main; filed with the reference number 122/12, at April, 16, 2012). The trial study protocol and a confirmation of the ethical review board stating adherence to the registered trial protocol are attached as S1 and S2 Figs. This study was registered after patient recruitment began, because only the approval of the Research Ethics Committee was necessary to conduct the study at our institution and due to an unintentional administrative delay. The authors confirm that all ongoing and related trials for this drug are registered. Written informed consent was obtained from all patients or their legal representatives. After obtaining written informed consent, 30 consecutive patients were enrolled into the study. All patients in the ICU were screened on a daily basis between 06:30 and 07:30 am for severe sepsis (sepsis-2 definition). Patients needed to fulfill two or more of the following SIRS criteria: (a) core temperature of >38°C or <36°C, (b) heart rate of >90 beats/minute, (c) respiratory rate of >20 breaths/minute or partial pressure of arterial carbon dioxide (PaCO2) <32 mmHg (all patients screened were breathing spontaneously, though intubated) or (d) total WBC absolute count >12,000 cells/mm^3^ or <4,000 cells/mm^3^. Additionally, severe sepsis was diagnosed, when microbiological results revealed microorganism growth in blood/sterile sites or when infected tissue was detected clinically (i.e., pneumonia) and at least one criteria for organ dysfunction was fulfilled [19]. After the enrollment of 15 patients, the IgM-IVIg application (Pentaglobin^®^, Biotest, Dreieich, Germany) was integrated into the unit’s SOP as treatment for patients with severe sepsis/septic shock, which generated two groups: patients before (Control) and after (IgM-IVIg) the implementation of the new SOP.

Patients were not eligible for this study if one or more of the following items applied: age <18 years, pregnancy, anticoagulation other than heparin or a known inherited coagulopathy or thrombophilia.

### Procedures

The parameters for the inclusion criteria and demographic data were recorded. Conventional laboratory analyses, including the INR, aPTT, platelet count, fibrinogen concentration, LBP, IL-6 levels, leukocyte count and microbiological results, were collected twice a day, at 04:00 am and 04:00 pm. Blood samples for the EAA, ROTEM^®^ and Multiplate^®^ analyses were collected on four consecutive days at 08:00 am, 02:00 pm and 08:00 pm. All blood samples were drawn from an existing arterial line and processed within one hour. For the EA, thrombelastometric and aggregometric measurements, EDTA-, citrated and heparinized tubes were obtained, respectively. EA levels were determined with the Endotoxin Activity Assay (EAA^™^, Spectral Diagnostics Inc., Toronto, ON, Canada), according to the manufacturer’s instructions. Thrombelastometry was performed using a ROTEM^®^ device (TEM International GmbH, Munich, Germany). Three tests were performed according to the manufacturer’s instructions, using 300 μl citrated bloods for each test. The tissue factor activated EXTEM-test, the tissue factor activated APTEM-test with added aprotinine for the detection of hyperfibrinolysis and the NATEM-test without clot activator with the addition of heparinase and CaCl_2_ only (20 μl heparinase and 0.2 M CaCl_2_) were utilized. Platelet function was determined by MEA using the whole blood impedance aggregometer Multiplate^®^ (Roche AG, Grenzach, Germany) based on impedance aggregometry as described by Cardinal and Flower [20]. For each test, 300 μl saline and 300 μl heparinized whole blood were pipetted into a temperature controlled test cell at 37°C. In vitro platelet aggregation was initiated by i) 0.5 mmol/l of arachidonic acid (ASPItest), ii) 6.4 mmol/l of ADP (ADPtest) and iii) 32 mmol/l of thrombin receptor activating peptide (TRAP-6, TRAPtest) using commercially available reagents. Platelet aggregability was calculated as the area under the aggregation curve (AUC), which was presented in arbitrary “aggregation units” (AU*min).

IgM-IVIg (Pentaglobin^®^, Biotest AG, Dreieich, Germany) was administered according to the manufacturer’s recommendation for three consecutive days, with a dose of 5 mg/kg/d over 12 hours (08:00 am to 08:00 pm) followed by a 12-hour pause.

### Statistical analysis and sample size

The presented study had an explorative nature. Data evaluating the possible effect of IgM-IVIg treatment on EA were not available. Therefore, no sample size calculation was performed for the repeated measurements. The sample size of 30 used in the present study was calculated for Gaussian distributed endpoints to generate confidence intervals with reasonable accuracy.

Statistical analyses were performed using SigmaStat 3.5 and SigmaPlot 12 (Systat Software GmbH, Erkrath, Germany) as well as SAS^®^ (SAS Institute GmbH, Heidelberg, Germany). Data was analyzed using a factorial repeated measures ANOVA with the parameters Pentaglobin, time and daily profile. For variables, which did not follow a normal distribution, rank-transformed data was used to apply this method. Specific post-hoc tests were performed, if statistically significant differences were detected in one of the methods mentioned above. Groups were compared using t-test, the Mann-Whitney-U test or the Fisher exact test analyses as appropriate. Depending on the results of the Kolmogorov-Smirnov-test, the results are given as the means (± SEM) or medians (interquartile range (IQR)).

## Results

**Fig 1:**
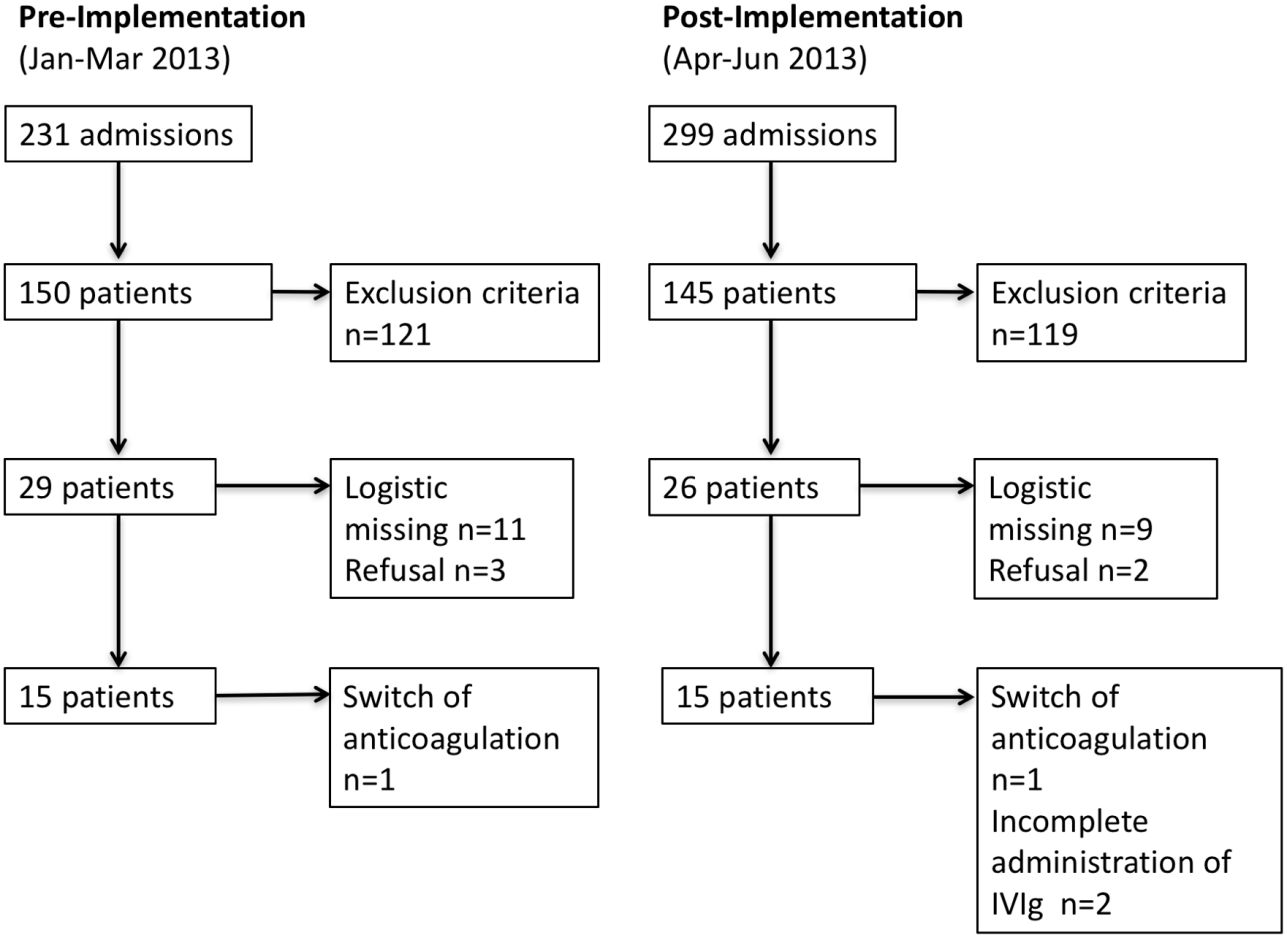
Patient Flow. Flow of patients before (Control; n = 14) and after (IVIg; n = 12) integrating the IgM-IVIg application into the unit’s SOP as a treatment for patients with severe sepsis.

All 30 patients fulfilled the criteria for severe sepsis or septic shock on enrollment and had been admitted to the ICU after trauma, major surgery or postoperative complications. Two patients were excluded from the study due to incomplete administration of IgM-IVIg and two patients were switched to an anti-coagulant other than heparin during the study period. The demographic and clinical characteristics of the remaining 26 patients are presented in Table 1, the flow of patients in Fig 1, respectively. Patients treated with IgM-IVIg had a significantly lower body mass index (BMI) with a median of 26.5 vs. 31.8 (p = 0.015) and suffered more often from diabetes (p = 0.03) and renal insufficiency prior to admission to the ICU (p = 0.019). APACHE II did not differ between the groups. On enrollment, n = 13 (92.8%) patients in the control group and n = 10 (83.3%) in the IgM-IVIg group fulfilled criteria for septic shock.

**Table 1:** Characterization of patients at baseline.

Variables	Control n = 14	IgM-IVIg n = 12	p-value*
Age [median (IQR)]	70 (63/76)	73 (67/77)	0.959
Male [n (%)]	10 (71.4)	8 (66.7)	0.67
BMI [median (IQR)]	31.8 (27.5/37)	26.5 (23.5/29.5)	0.015
APACHE II [median (IQR)]	35 (30/41)	35 (31/38)	0.69
Source of infection	Lung n = 5	Lung n = 3	
	Abdominal n = 5	Abdominal n = 4	
	Positive Blood Culture n = 2	Positive Blood Culture n = 1	
	Other n = 1	Other n = 2	
	No Pathogen n = 1	No Pathogen n = 2	
Diabetes [n (%)]	5 (35.7)	0 (0)	0.03
Arterial Hypertension [n (%)]	8 (57.1)	8 (66.7)	0.464
Cardiovascular Disease [n (%)]	4 (28.6)	7 (58.3)	0.128
Renal Insufficiency [n (%)]	9 (64.3)	2 (16.7)	0.019
Hepatic Insufficiency [n (%)]	3 (21.4)	1 (8.3)	0.359
COPD [n (%)]	4 (28.6)	1 (8.3)	0.213
Nicotine Abuse [n (%)]	3 (21.4)	1 (8.3)	0.359
Immunosuppression [n (%)]	1 (7.1)	0 (0)	0.538

BMI, body mass index; APACHE II, Acute Physiology And Chronic Health Evaluation II; COPD, Chronic Obstructive Pulmonary Disease; IQR, interquartile range; IgM-IVIg, IgM-enriched intravenous immunoglobulin

* p-values were calculated using the Fisher exact test, Student’s t-test or the Mann-Whitney-test as appropriate, values <0.05 were considered statistically significant

### EA levels

**Fig 2:**
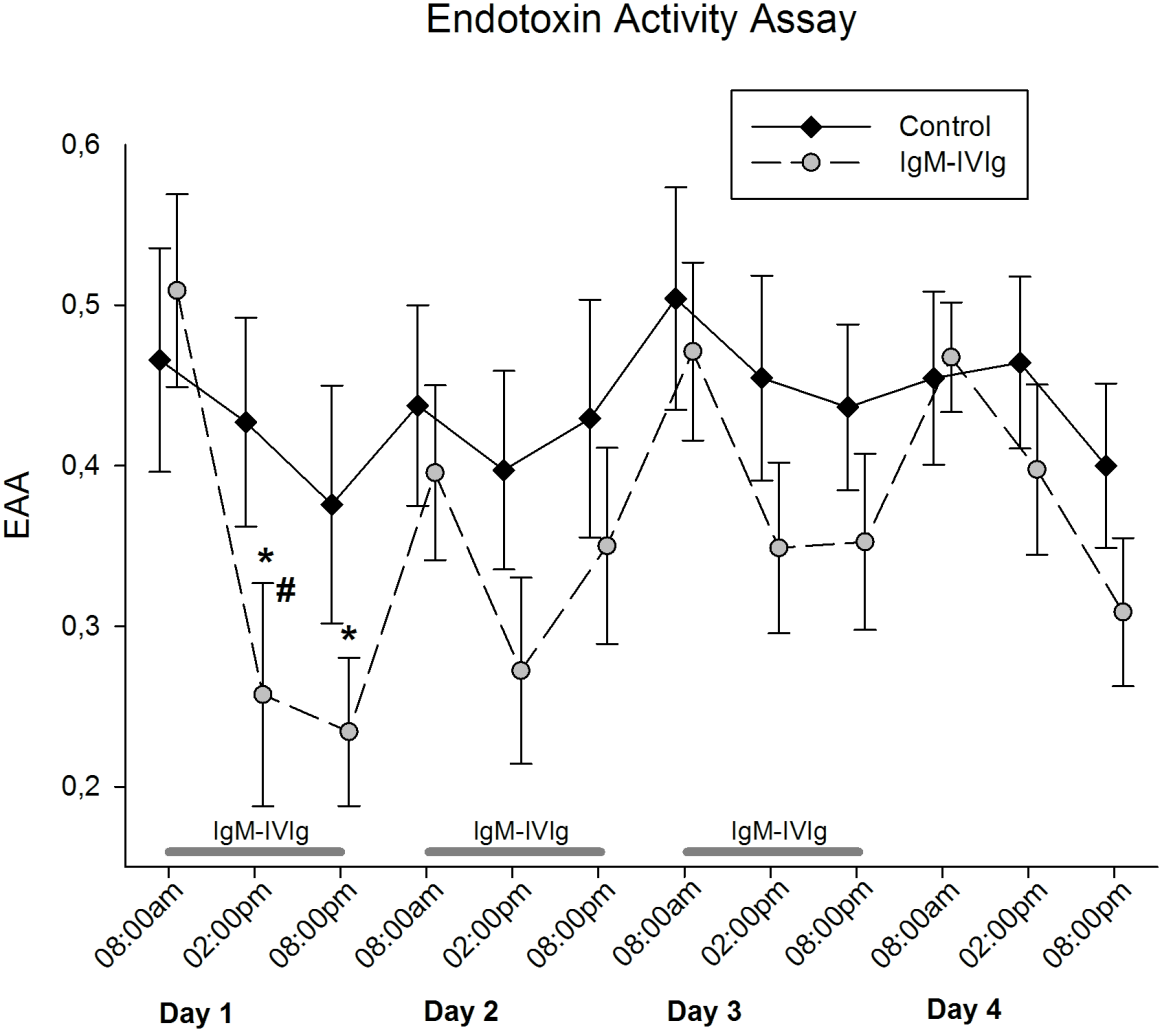
Endotoxin Activity Assay. Endotoxin activity (EA) was measured three times a day during the four-day observational period and treatment with IgM-enriched immunoglobulin (IgM-IVIg); *, p = 0.01 and p = 0.003, respectively demonstrate the significantly decreased EA in the IgM-IVIg group on day 1; #, p = 0.04 denotes the significant difference between the IgM-IVIg and the control groups, as calculated using Student’s t-test.

The EA levels on enrollment did not differ between the control and IgM-IVIg groups (0.51 ± 0.06 vs. 0.47 ± 0.07). On day 1, patients receiving IgM-IVIg showed a significant reduction in EA following 6 and 12 hours of treatment (0.26 ± 0.07, p = 0.01 and 0.25 ± 0.04, p = 0.003 (Fig 2)). The EA levels on day 1 following 6 hours of treatment were significantly reduced in the IgM-IVIg group compared with the control group (0.26 ± 0.07 vs. 0.43 ± 0.07, p = 0.04 (Fig 2)). No differences in EA levels either within or between the groups were observed during the following time-course. Regardless of the group, the course of EA during the day tended to be higher in the morning and lower at 02:00 pm and 08:00 pm.

### Conventional coagulation laboratory measurements

**Fig 3:**
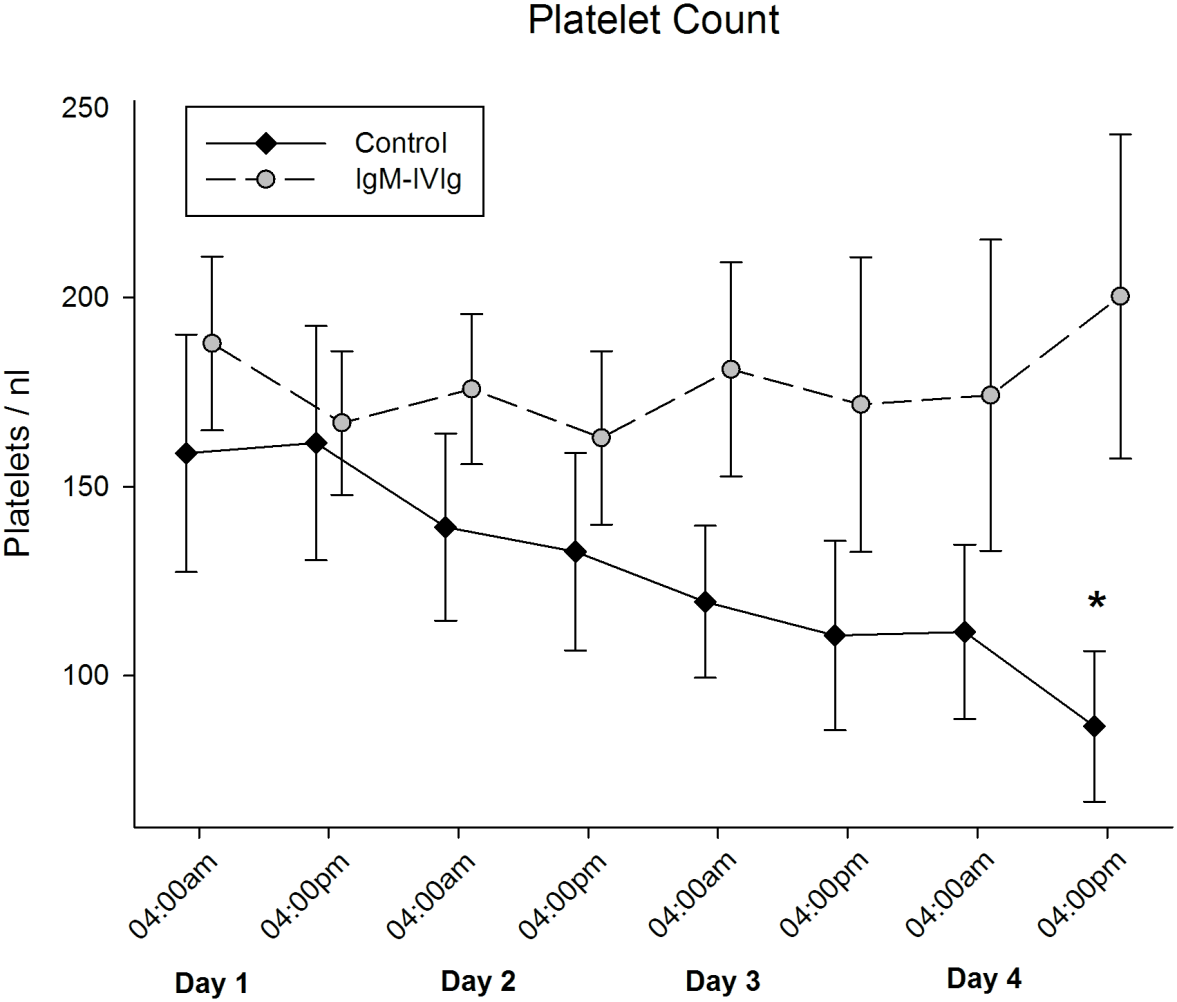
Platelet Count. Platelet count was measured two times a day during the four-day observational period and treatment with IgM-enriched immunoglobulin (IgM-IVIg); *, p = 0.016 indicates the significant difference between the groups at the end of the observational period, as calculated using Student’s t-test.

The mean platelet count on enrollment did not differ between the groups (159/nl ±31 vs. 188/nl ±23 (p = 0.478)). During the four-day treatment course, the platelet count in the control group continuously declined, resulting in significantly lower values on day four compared with the IgM-IVIg group (87/nl ±20 vs. 200/nl ±43 (p = 0.016)) (Fig 3).

**Fig 4:**
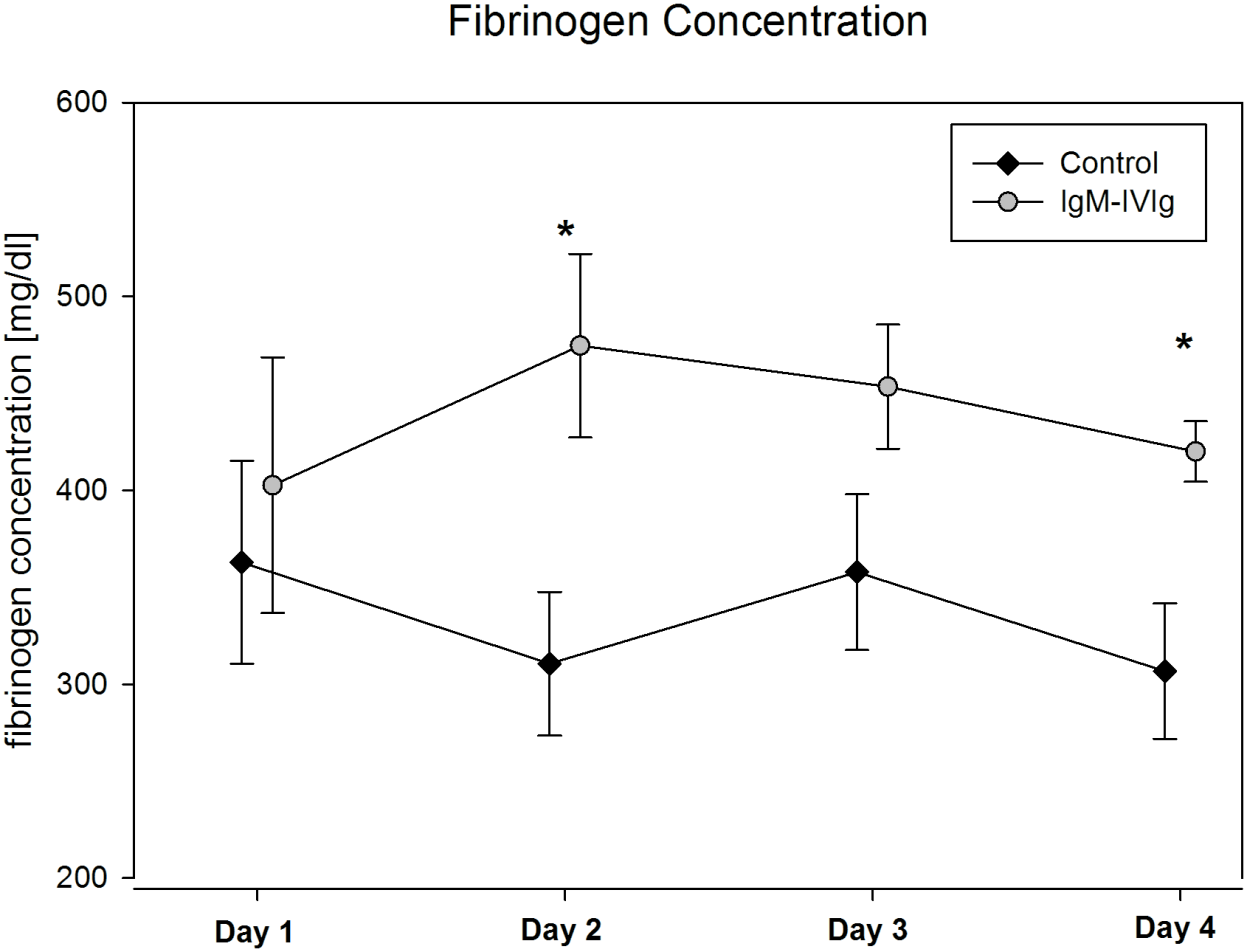
Fibrinogen Concentration. The fibrinogen concentration was measured once a day during the four-day observational period and treatment with IgM-enriched immunoglobulin (IgM-IVIg); * indicates a significant difference between the groups on day 2 (p = 0.016) and day 4 (p = 0.017), as calculated using Student’s t-test.

The fibrinogen concentration on enrollment did not differ between the groups (363 mg/dl ±52 vs. 403 mg/dl ±66 (p = 0.64)). During the following days, the fibrinogen concentration was lower in the control group, with a significant difference being observed on day 2 (311 mg/dl ±37 vs. 475 mg/dl ±47 (p = 0.015)) and day 4 (307 mg/dl ±35 vs. 420 mg/dl ±16 (p = 0.017)) (Fig 4).

None of the other conventional laboratory measurements, including conventional clotting variables, such as INR, aPTT or prothrombin time, differed between the groups at any time.

### ROTEM^®^

There was no difference between the groups with respect to the viscoelastic ROTEM variables in the extrinsically activated EXTEM-test. The APTEM-test showed no evidence for hyper-fibrinolysis in the enrolled patients. The median values of the viscoelastic parameters in the NATEM-test tended to be more procoagulatory in the IgM-IVIg group during the complete observational period of four days; however, they were not significantly different from the control values (Table 2). This tendency persisted during the observation period without a statistically significant difference between the groups.

**Table 2:** Viscoelastic NATEM variables.

Parameter	Group	Day 1	Day 2	Day 3	Day 4
CFT (sec) [Median (IQR)]	Control	299 (198/654)	408 (279/647)	387 (271/591)	328 (229/546)
	IgM-IVIg	238 (147/367)	173 (125/286)	201 (147/306)	177 (143/276)
CT (sec) [Median (IQR)]	Control	900 (681/1084)	1051 (847/1358)	1046 (903/1082)	953 (840/1137)
	IgM-IVIg	697 (552/981)	724 (615/1185)	839 (613/1218)	788 (660/1334)
α-angle (°) [Median (IQR)]	Control	44 (27/55)	34 (24/48)	37 (27/47)	46 (31/57)
	IgM-IVIg	58 (42/65)	61 (48/67)	57 (44/64)	61 (50/65)
MCF (mm) [Median (IQR)]	Control	53 (41/67)	54 (42/66)	52 (42/62)	47 (31/62)
	IgM-IVIg	63 (58/69)	63 (58/69)	67 (59/72)	71 (63/75)

CFT, clot formation time; CT, clotting time; MCF, mean clot firmness; IQR, interquartile range; IgM-IVIg, IgM enriched intravenous immunoglobulin; Control group n = 14, IgM-IVIg group n = 12 (days 1 and 2), n = 11 (day 3) and n = 10 (day 4); no significant differences between the groups

### Multiplate^®^

There were no significant differences between the groups with respect to aggregometric parameters in the ASPI-test, ADP-test or TRAP-test (data not shown).

### Inflammatory markers

With the exception of LBP, none of the determined inflammatory markers (IL-6 and leukocyte count) showed statistically significant differences between the groups at baseline. Additionally, the markers did not differ during the subsequent time-course. Data are shown in Table 3.

**Table 3:** Inflammatory Markers.

Parameter	Group	Day 1	Day 2	Day 3	Day 4
LBP [μg/l] [Median (IQR)]	Control	23.3 * (19.3/29.5)	36.2 (19.1/53.2)	26.5 (19.2/40.7)	19.2 (15.4/26.5)
	IgM-IVIg	41.1 * (38.4/46.3)	36.1 (33.5/51.2)	32.0 (24.1/33.3)	28.0 (16.6/31.8)
IL-6 [pg/μl] [Median (IQR)]	Control	312.5 (22.2/1850.5)	422.6 (73.9/1033.2)	142.9 (70.1/674.7)	75.9 (42.5/331.5)
	IgM-IVIg	478.6 (159.2/1025.4)	322.4 (164.7/805.7)	125.4 (88.9/541.3)	99.5 (80.3/529.0)
Leukocytes [/nl] [Median (IQR)]	Control	15.1 (8.6/20.7)	15.6 (9.8/24.0)	15.5 (12.0/28.3)	13.7 (10.4/19.6)
	IgM-IVIg	15.2 (7.1/23.2)	15.5 (10.6/16.3)	15.4 (9.7/20.0)	11.7 (7.6/16.2)

LBP, lipopolysaccharide binding protein; IL-6, interleukin-6; IQR, interquartile range; IgM-IVIg, IgM enriched intravenous immunoglobulin; Control group n = 14, IgM-IVIg group n = 12 (days 1 and 2), n = 11 (day 3) and n = 10 (day 4);

* indicating p = 0.034, as calculated using the Mann-Whitney-test

### SOFA Scores and Organ Support

The SOFA score time-course data and required organ support are shown in Table 4. No significant differences between or within the groups were detected.

**Table 4:** Scores and Clinical Information on Organ Support.

		Day 1	Day 2	Day 3	Day 4
SOFA	Control	10.6 ±1.2	9.8 ±1.4	10.1 ±1.4	9.5 ±1.6
	IgM-IVIg	11.7 ±0.9	10.3 ±0.8	9.4 ±1.2	7.0 ±1.8
Vasopressor Therapy [n (%)]	Control	13 (92.8)	11 (78.6)	10 (71.4)	8 (57.1)
	IgM-IVIg	10 (83.3)	10 (83.3)	5 (45.4)	4 (40.0)
Mechanical Ventilation [n (%)]	Control	13 (92.8)	13 (92.8)	11 (78.6)	11 (78.6)
	IgM-IVIg	12 (100.0)	10 (83.3)	8 (72.7)	6 (60.0)
Dialysis [n (%)]	Control	5 (35.7)	7 (50.0)	9 (64.3)	9 (64.3)
	IgM-IVIg	4 (33.3)	4 (33.3)	5 (45.4)	4 (40.0)

SOFA, Sequential Organ Failure Assessment; IgM-IVIg, IgM-enriched intravenous immunoglobulin; Control group n = 14, IgM-IVIg group n = 12 (day 1 and 2), n = 11 (day 3) and n = 10 (day 4); no significant differences between the groups were detected using the Students’ t-test or Fisher Exact test

## Discussion

The aim of this study was to investigate a potential effect of IgM-enriched immunoglobulin (Pentaglobin^®^) on the EA levels of patients with severe sepsis. Additionally, we investigated the effects of an IgM-IVIg therapy on the functional coagulation parameters in thrombelastometric and aggregometric analyses and on the conventional coagulation parameters. Furthermore, we examined its effects on inflammatory parameters, such as LBP, IL-6 and leukocyte count.

We observed a significant reduction in the EA levels over the first day of IgM-IVIg application in the IgM-IVIg group. Compared with the control group, the EA levels were significantly lower at 6 hours of treatment. During the following three days of observation, there was no further significant difference between the groups; however, the EA levels tended to be lower in the IgM-IVIg group. Because the effect of IgM-IVIg therapy on EA has not previously been evaluated, interpreting these findings seems difficult. The dosages on the second and third days of treatment might have been insufficient due to either a saturation effect or an increase in EA after antibiotic treatment induction. Because a less marked daily drop in EA was also observed in the control group, there might also be a circadian effect on the EA in addition to possible effects from the IgM-IVIg treatment.

The effects of IgM-IVIg on the EA levels following endotoxin application are mostly described for animal models when endotoxin and IgM-IVIg were administered in close succession and with short observational periods [7, 21, 22]. Most previous studies in humans focused on neonates and preterm infants, who differ in their immune responses from adults, or on neutropenic patients [8, 23, 24]. Studies evaluating adults with severe sepsis or septic shock found differences in clinical outcomes and parameters without measuring the endotoxin levels during the IgM-IVIg treatment [25, 26].

Behre et al. discriminated non-responders from responders in septic patients with neutropenia due to hematologic malignancies based on their endotoxin levels after receiving a treatment with IgM-IVIg [8]. In the present study, patients were not evaluated with respect to a potential non-response to the IgM-IVIg treatment. Therefore, our results could be influenced by non-responders within the groups.

Despite observing a reduction in EA levels in the IgM-IVIg group on the first day, we did not find a significant difference in the viscoelastic or aggregometric measurements (EXTEM, APTEM, NATEM, or ASPI-, ADP- and TRAP-tests) between the groups on any of the observed days. Endotoxin not only triggers innate immunity in terms of activating monocytes, macrophages and granulocytes, resulting in a release of various cytokines, but systemic endotoxin also leads to an increase in circulating tissue factor. In combination with FVIIa, tissue factor activates FIX and FX, thereby leading to thrombin formation [27]. In patients with early SIRS or sepsis, the EA levels correlate with the viscoelastic parameters (e.g., CT, CFT, MCF and Alpha angle) in the NATEM-test [18]. However, we included only patients who had already developed severe sepsis and septic shock, thereby presumably selecting patients, who suffered from an ongoing systemic inflammation for a longer period of time. For example, the procoagulatory phase that is associated with early stages of systemic inflammation probably tended to the condition of disseminated intravascular coagulation in some of the patients, which has been demonstrated to lead to rather anticoagulatory effects on thrombelastometric parameters [28, 29]. One could further speculate that in the progressed stages of sepsis, changes in the coagulation system are already profound and, thus, that a mere elimination of endotoxin is not sufficient to reverse the effects on viscoelastic variables in patients with severe sepsis or septic shock.

At baseline, the APACHE II and SOFA scores and the inflammatory markers showed no significant differences between the groups, except for the LBP baseline measurement. Furthermore, during the observational period, no differences between or within the groups could be observed. Therefore, we did not consider different severity stages of sepsis during the observational period. The standard coagulation measurements differed significantly in terms of both the fibrinogen concentration and the platelet count course during the four-day observation period. Both parameters have been shown to decrease after exposing rodents to endotoxin [12, 30]. With platelet counts staying stable over time in the IgM-IVIg group, one could speculate that the IgM-IVIg therapy might affect sepsis-associated thrombocytopenia. In an animal model, Hofmann et al. found a reduced drop in platelet count after the simultaneous application of endotoxin and IgM-IVIg in hamsters [22]. Irrespective of the cause, thrombocytopenia or a drop in platelet count of 30% or more in critically ill patients are independent predictors of mortality in ICUs [31, 32]. In addition to their hemostatic function, platelets have an active part in the immune response, in that they are capable of secreting a large variety of proteins involved in inflammation [33, 34].

Reduced platelet activation could also explain the difference in fibrinogen concentrations seen between the groups, because platelet activation leads to a consumption of fibrinogen due to clot formation. Our data could suggest that the effect of IgM-IVIg might be due to an attenuation of endotoxin-mediated platelet activation and the subsequent induction of inflammation and clot formation and not to the mere elimination of endotoxin from the blood stream.

The present study had only an explorative and descriptive nature and did not evaluate clinical outcome of the patients. The results presented must be interpreted carefully due to the small sample size. Nevertheless, these findings might be the basis for future studies targeting IgM-IVIg as a therapeutic option in patients with high EA levels. We found hints that IgM-IVIg attenuates EA levels, with potential effects on sepsis-related coagulopathy.

## Conclusion

Treatment with IgM-enriched IVIg (Pentaglobin^®^) attenuates the EA levels in patients with severe sepsis and septic shock. Furthermore, both the platelet count and fibrinogen concentrations were higher in the IgM-IVIg group than in controls, whereas neither viscoelastic or aggregometric measurements nor inflammatory markers, such as LBP levels, IL-6 levels or leucocyte counts, were influenced by treatment with IgM-IVIg.

## Supporting Information

S1 Fig Trial Study Protocol. (PDF)

S2 Fig Statement of the Ethical Review Board. Statement of the ethical review board confirming that the study as it was conducted adhered to the trial study protocol as it was approved.
(PDF)

S3 Fig TREND Checklist. (PDF)
