# Supplementary material for: IgM-Enriched Immunoglobulin Attenuates Systemic Endotoxin Activity in Early Severe Sepsis: A Before-After Cohort Study
Source: PLoS One. 2016 Aug 9;11(8):e0160907. doi: 10.1371/journal.pone.0160907 (PMC4978476; doi:10.1371/journal.pone.0160907)
Supplement: S1 Fig — (PDF) [file pone.0160907.s001.pdf]

Diagnostic Monitoring of Endotoxemic Patients with the Endotoxin Activity Assay (EAA®) receiving Pentaglobin (DEEP trial)

1. Investigators

- Principal Investigator:

Prof. Dr. Dr. Kai Zacharowski, FRCA
Klinik für Anästhesiologie, Intensivmedizin und Schmerztherapie,
Klinikum der Johann Wolfgang Goethe – Universität Frankfurt am Main

- Further investigators:

Dr. Tobias Bingold
Dr. A. Koch
Dr. F. Jäger
Dr. C. Weber
Dr. M. Klages
PD. Dr. P. Meybohm
Klinik für Anästhesiologie, Intensivmedizin und Schmerztherapie,
Klinikum der Johann Wolfgang Goethe – Universität Frankfurt am Main

2. Background

In Germany, 79,000 patients are diagnosed with sepsis annually. Another 75,000 patients progress into the more severe stages "severe sepsis" and "septic shock" [1]. The mortality in patients with septic shock remains very high with up to 54%-60% [1, 2]. The definition of sepsis is "systemic inflammation induced by an infection". In most cases bacteria are responsible for the cause of the infection, which gain access to the bloodstream of the patients. Bacteria can generally be divided into Gram-positive and Gram-negative pathogens. Approximately 25%-30% of all septic patients are associated with Gram-negative bacteria [3]. All Gram-negative bacteria share one similarity: the presence of endotoxins on the bacterial surface, which has a strong inflammatory impact on the immune system of the infected patients. These endotoxin levels in the blood of the patients correlate with patient mortality [4].
The human polyspecific immunoglobulin preparation Pentaglobin is approved for treating severe bacterial infections concomitantly with antibiotic therapy. One of the major mode of action of Pentaglobin is to neutralize bacterial endotoxins. This is especially ascribed to the presence of the immunoglobulin class M (IgM) (immunoglobulin class composition of Pentaglobin: 12% IgM, 76% IgG und 12% IgA) [5]. The Pentaglobin mediated neutralization activity of endotoxins has been demonstrated in *ex vivo* experiments [6, 7] and in a randomized controlled clinical trial [8]. In the latter, endotoxin concentrations were determined with the limulus test. Nowadays, a novel bedside test system is available. The so called "Endotoxin Activity Assay" (EAA®) allows a determination of endotoxin levels in patient blood within 30 minutes [9]. Results are stated as Endotoxin Activity (EA) units with values between 0 (minimal) and 1 (maximal). Three EA intervals were empirically evaluated: <0.4 (low),

0.4-0.6 (intermediate) and >0.6 (high). These ranges correlate with the severity of sepsis, dosages of vasopressors and clinical outcome [10, 11].

Sepsis has diverse consequences for the organism. One major issue of sepsis is the dysbalance of the pro- and anticoagulation system. Procoagulatory factors, such as tissue factor (TF) and thrombin are much more activated, and contrarily the activity of anticoagulatory factors, such as antithrombin, protein C and tissue factor pathway inhibitor, are decreased. This coagulation pathology results in a disseminated intravascular coagulation (DIC), which goes hand in hand on one side with microvascular thrombi and fibrin deposits and on the other side with a diffuse bleeding tendency [12]. Microvascular thrombi in capillaries play an important role in development of perfusion failure, which can subsequently lead to multiorgan failure.
Coagulation tests such as rotation thromboelastometry (ROTEM®) have gained importance in intensive care medicine. ROTEM® analysis provides useful information for the clinicians within minutes, if an existing bleeding tendency is due to a lack of coagulation factors in general, a lack of fibrinogen, hyperfibrinolysis, or a combination of all. Interestingly, the ROTEM®-derived clotting time (CT) decreases, if blood is contaminated with endotoxin before or during a rotation thromboelastometry measurement [13, 14]. A clinical study with human volunteers suggested, that several ROTEM® parameters are affected by a systemic endotoxin administration [15]. The integration of an endotoxin filter in a heart-lung-machine could increase the clot stability in an interventional study with cardiac surgical patients [16]. Rotation thromboelastometry may have the potential to monitor indirectly endotoxin activity and thereby severity of sepsis, additionally to the commercially available EAA®. Very recently, Adamzik et al. showed that rotation thromboelastometric parameters such as clotting formation time (CFT), maximum clot firmness (MCF) and alpha angle may have a even better predictive value regarding 30-day mortality in patients with severe sepsis compared to standard scoring systems, e.g. the simplified acute physiology score (SAPS II) or the sequential organ failure assessment (SOFA) [17].
In this context, Moreau et al. revealed, that a 30% drop in platelet count independently predicts death in critical ill patients [18]. It seems that both, platelet trapping in capillaries and their subsequent plugging during sepsis are responsible for thrombocytopenia. This was demonstrated by intravital microscopy in septic mice [19]. However, not only a decrease in platelet count is featuring sepsis, also platelet function is impaired. Gawaz et al. demonstrated that plasma from patients with severe sepsis sensitizes normal platelets to ‘over’-respond to ADP-stimulation *in vitro* [20]. Enhanced platelet activation, resulting in aggregation, platelet-endothelial and platelet-leukocyte interaction, is affiliated to reactive oxygen species [21,22,23]. It seems likely, that other platelet receptors and glykoproteins are involved in the alteration of platelet function during endotoxemia. Therefore, platelet function will be investigated by stimulation tests in impedance aggregometry (MULTIPLATE®).

This Investigator Initiated Trial is intended to answer the following questions:

- Does the EAA® test reflect attenuation / neutralization of blood endotoxin activity after Pentaglobin therapy *in vivo*?
- If yes, what is the impact of routinely administered Pentaglobin therapy on endotoxin blood levels in septic patients?
- Does rotation thromboelastometry (ROTEM®) and impedance aggregometry (Multiplate®) reflect attenuation / neutralization of endotoxin activity after Pentaglobin therapy *in vivo*?

- If yes, what is the impact of routinely administered Pentaglobin therapy on the coagulation parameters and platelet function in septic patients?

3. Hypothesis

It is expected that the EAA® test detects changes in endotoxin blood levels in septic patients receiving Pentaglobin during the observation period.

4. Endpoints

- **Primary endpoint**
    - Time course of endotoxin blood levels (EAA® test) in patients with proven or suspected severe sepsis, focusing on Pentaglobin related changes

- **Secondary endpoints**
    - Focus, cause of sepsis
    - Microbiological results
    - In-hospital mortality
    - ICU mortality
    - ROTEM® and Multiplate® assays
        - ROTEM® : NATEM®, EXTEM®, HEPTEM®, APTEM®
        - Multiplate®: TRAPtest®, ASPItest®, ADPtest®
    - Quick, thromboplastin time, platelet count

5. Design

- **Informed Consent**

Patients will be included in this observational study following oral and written information and written consent. The form for obtaining primary consent from awake and orientated patients is attached to this application.
However, the majority of the patients, who will be included in this study, will not be in a position to understand and sign the written consent due to their sever illness and/or their critical care therapy. If no representative is nominated or available, consent will be obtained from the patient as soon as the patient regains his/her capabilities. The form for obtaining secondary consent from former analog-sedated/not-orientated patients is attached to this application.
If a legal representative is available and contactable, this person decides on his/her own responsibility on behalf of the patient. The information and consent process will then be carried out in the same way with the representative as with the patient. The relationship between the representative and the patient has to be declared handwritten on the written consent form. If the representative is not personally available, he/she may also give his/her consent by fax or in exceptional cases initially on the telephone. If the consent

was given by fax or telephone, the original signature has to be obtained as soon as possible. The consent form for legal representatives is attached to this application.
If the patient is not able to give consent for a longer time, a legal representative needs to be found and give his/her consent as soon as possible.
If either the patient or the representative deny their consent, the patient has to be excluded from this observational trial from the time of denial.
If the patient dies during the course of this observational trial before a written consent could be obtained, it will be assumed that the patient gave his/her consent.

- **Collection of the following parameters (observational)**

    - Age, height, weight and sex of the patient
    - Septic shock criteria
    - Proof of infection
    - APACHE II score
    - SAPS III score
    - SOFA score
    - Form/Dosage of prophylactic/therapeutic anticoagulation
    - Need for substitution of coagulation factors and other hemostatic therapy (e.g. DDAVP, Tranexamic acid)
    - Need for transfusion
    - Routine lab
        - Hemogram
        - Creatinine
        - Liver enzymes
        - PCT
        - IL-6
        - CRP
        - Platelet count
        - PTT, Quick
        - LBP
    - Duration of Pentaglobin administration each day
    - ROTEM®, Multiplate® and EAA® test results
    - Focus, cause of sepsis
    - Microbiological results
    - In-hospital mortality (will be retrieved from patient record)
    - ICU mortality (will be retrieved from patient record)

- **Conducted measurements and time points**

All patients admitted to the ICU will be screened for severe sepsis daily between 06:30 and 07:30 am. If the criteria for severe sepsis are fulfilled, arterial blood will be withdrawn via the in situ in-dwelling catheter from patients (1 x 2.7 ml in a EDTA tube) to perform the EAA® test. Two additional arterial blood sample will be withdrawn via the in situ in-dwelling catheter (1 x 3.0 ml in a citrate tube) to perform the ROTEM® assay and (1 x 2.0 ml in a

heparin tube) to perform the Multiplate® assay. During the observation period (four consecutive days), three blood samples (1 x 2.7ml, 1 x 3.0ml and 1 x 2.0 ml) will be withdrawn via the in situ in-dwelling catheter every 6 hours after the inclusion of the patient to perform the EAA® test, ROTEM® and Multiplate® assays. Initiation of Pentaglobin therapy is based on consultant's decision, made by clinical considerations, and performed according to product information.

6. Statistics

- **Number of patients to be included**

  30 patients with severe sepsis. It is assumed, that 10-15 Patients will receive Pentaglobin therapy.

- **Biometrical analysis**

  This is an explorative trial. The primary endpoint (change of the endotoxin blood levels during the observation period) as well as most secondary endpoints will be analyzed with a suitable 95% parametric or non-parametric confidence intervals. For Gaussian distributed endpoints, a sample size of 30 leads to confidence intervals with a total length of less than the order of magnitude of the standard deviation. This seems a reasonable accuracy for this explorative analysis.

  Furthermore, Pearson correlation or Spearman correlation coefficients will be used to explore correlations between markers and markers at different observation times. Further explorative tests may be performed as appropriate. All tests will be two-sided with a significance level of α=5%.

  In addition, descriptive statistics will be calculated.

7. Duration

Approximately 3-4 patients per month are diagnosed with severe sepsis. A study duration of approximately 8-10 months is planned for this observational trial.

8. Inclusion and exclusion criteria

- Inclusion criteria
  - Written consent of the patient or legal person in charge
  - Patients with suspected or proven severe sepsis
  - Age ≥ 18 years

- Exclusion criteria
    - o Pregnancy
    - o Anticoagulative therapy other than heparin
    - o Inherited coagulopathy or thrombophilia

9. How will the target values be determined?

The blood sample will directly be measured in the corresponding bedside tests after blood withdrawal:

- o 1 x 2.7 ml EDTA tube (→ EAA® test)
- o 1 x 3.0 ml citrate tube (→ ROTEM®)
- o 1 x 2.0 ml heparin tube (→ Multiplate®)

The secondary endpoints will be documented daily for each included patient.

10. Evaluation

The results of this prospective non-interventional, observational investigator initiated trial will be analyzed according to biometrical standards.

11. Data protection

The written consent includes the allowance to save the collected data pseudonymized and to use it in anonymous format for scientific publications. The collected data will be treated confidentially without the submission to third parties.

12. Insurance

Patients insurance is not intended

References:

1. Engel C, Brunkhorst FM, Bone HG, Brunkhorst R, Gerlach H, Grond S, Gruendling M, Huhle G, Jaschinski U, John S et al.: Epidemiology of sepsis in Germany: results from a national prospective multicenter study. *Intensive Care Med* 2007, 33:606-618.
2. Brun-Buisson C: The epidemiology of the systemic inflammatory response. *Intensive Care Med* 2000, 26 Suppl 1:S64-S74.
3. Annane D, Aegerter P, Jars-Guincestre MC, Guidet B. Current epidemiology of septic shock: the CUB-Rea Network. *American Journal of Respiratory Critical Care Medicine* 2003; 1687: 165-172.
4. Thomson AP, Hart A, Sills JA, Harris F: Anti-endotoxin therapy for fulminant meningococcal septicemia– Pilot study. Pediatric Rev. Commun 1991; 5: 199–205.
5. Trautmann M, Held TK, Susa M, Karajan MA et al.: Bacterial lipopolysaccharide (LPS)-specific antibodies in commercial human immunoglobulin preparations:

superior antibody content of an IgM-enriched product. *Clinical and Experimental Immunology* 1998; 111: 81–90.
6. Berger D, Schleich S, Seidelmann M, Berger HG: Antiendotoxic therapy with polyclonal and polyvalent immunoglobulins: in vitro and in vivo studies. In: Faist E Meakins JL, Schildberg FW (eds) *Host Defense Dysfunction in Trauma, Shock and Sepsis*. Springer-Verlag 1993, Heidelberg, pp 1164–1174.
7. Oesser S, Schulze C, Seifert J: Protective capacity of an IgM/IgA-enriched polyclonal immunoglobulin-G preparation in endotoxemia. Res. Exp. Med. 1999; 198: 325–339.
8. Schedel I, Dreikhausen U, Nentwig B, et al.: Treatment of Gram-negative septic shock with an immunoglobulin preparation: A prospective, randomized clinical trial. Crit Care Med 1991; 19: 1104–1113.
9. Marshall JC, Walker PM, Foster DM, Harris D, Ribeiro M, Paice J, Romaschin AD, Derzko AN: Measurement of endotoxin activity in critically ill patients using whole blood neutrophil dependent chemiluminescence. *Crit Care* 2002, 6:342-348.

10. Monti G, Bottiroli M, Pizzilli G, Minnini M, Terzi V, Vecchi I, Gesu G, Brioschi P, Vesconi S, Casella G: Endotoxin activity level and septic shock: a possible role for specific anti-endotoxin therapy? *Contrib Nephrol* 2010, 167:102-110.

11. Marshall JC, Foster D, Vincent JL, Cook DJ, Cohen J, Dellinger RP, Opal S, Abraham E, Brett SJ, Smith T et al.: Diagnostic and prognostic implications of endotoxemia in critical illness: results of the MEDIC study. *J Infect Dis* 2004, 190:527-534.

12. Levi M, van der PT: Inflammation and coagulation. *Crit Care Med* 2010, 38:S26-S34.

13. Hartmann M, Ozlugedik S, Peters J: Thiopental inhibits lipopolysaccharide-induced tissue factor expression. *Anesth Analg* 2009, 109:109-113.

14. Zacharowski K, Sucker C, Zacharowski P, Hartmann M: Thrombelastography for the monitoring of lipopolysaccharide induced activation of coagulation. *Thromb Haemost* 2006, 95:557-561.

15. Spiel AO, Mayr FB, Firbas C, Quehenberger P, Jilma B: Validation of rotation thrombelastography in a model of systemic activation of fibrinolysis and coagulation in humans. *J Thromb Haemost* 2006, 4:411-416.

16. Blomquist S, Gustafsson V, Manolopoulos T, Pierre L: Clinical experience with a novel endotoxin adsorbtion device in patients undergoing cardiac surgery. *Perfusion* 2009, 24:13-17.

17. Adamzik M, Langemeier T, Frey UH, Gorlinger K, Saner F, Eggebrecht H, Peters J, Hartmann M: Comparison of thrombelastometry with SAPS II and SOFA scores for the prediction of 30-day survival: a cohort study. *Shock* 2011, 35:339-42.

18. Moreau D, Timsit JF, Vesin A, Garrouste- Orgeas M, de Lassence A, Zahar JR, Adrie C, Vincent F, Cohen Y, Schlemmer B, Azoulay E. Platelet count decline: an early prognostic marker in critically ill patients with prolonged ICU stays. Chest 131: 1735–1741, 2007.
19. Secor D, Li F, Ellis CG, Sharpe MD, Gross PL, Wilson JX, Tyml K. Impaired microvas- cular perfusion in sepsis requires activated coagulation and P-selectin-mediated platelet adhesion in capillaries. Intensive Care Med 36: 1928–1934, 2010.
20. Gawaz M, Dickfeld T, Bogner C, Fateh-Moghadam S, Neumann FJ. Platelet function in septic multiple organ dysfunction syndrome. Intnesive Care Med 23: 379-385, 1998
21. Wu F, Wilson JX, Tyml K. Ascorbate inhibits iNOS expression and preserves vasoconstrictor responsiveness in skeletal muscle of septic mice. Am J Physiol Re-gul Integr Comp Physiol 285: R50–R56, 2003.

22. Krotz F, Sohn HY, Pohl U. Reactive oxy- gen species: players in the platelet game. Arterioscler Thromb Vasc Biol 24: 1988– 1996, 2004.
23. Stef G, Csiszar A, Ziangmin Z, Ferdi- nandy P, Ungvari Z, Veress G. Inhibition of NAD(P)H oxidase attenuates aggrega- tion of platelets from high-risk cardiac patients with aspirin resistance. Pharma- col Rep 59: 428–436, 2007.

Date – Signature **Prof. Dr. Dr. K. Zacharowski (*Principal Investigator)***
