# Supplementary material for: IgM-Enriched Immunoglobulin Attenuates Systemic Endotoxin Activity in Early Severe Sepsis: A Before-After Cohort Study
Source: PLoS One. 2016 Aug 9;11(8):e0160907. doi: 10.1371/journal.pone.0160907 (PMC4978476; doi:10.1371/journal.pone.0160907)
Supplement: S2 Fig — Statement of the ethical review board confirming that the study as it was conducted adhered to the trial study protocol as it was approved. (PDF) [file pone.0160907.s002.pdf]

GOETHE
UNIVERSITÄT
FRANKFURT AM MAIN

Zentrum der Pharmakologie

Institut für Klinische Pharmakologie
Direktor: Prof. Dr. Dr. G. Geißlinger

PLOS ONE
Managing Editor
Ms Iratxe Puebla

Prof. Dr. med. Sebastian Harder

Arzt für Klinische Pharmakologie

e-mail
Harder@em.uni-frankfurt.de

Telefon (Durchwahl)	*Telefax*	*Datum*
069/6301-6423	069/6301-83921	25.05.2016

PONE-D-16-07587: “IgM-enriched immunoglobulin attenuates systemic endotoxin activity in early severe sepsis: A Before-After Cohort Study”

Dear Ms Iratxe Puebla, dear editorial board,
Herewith I confirm that the protocol underlying the submitted manuscript stated above has been reviewed and approved pre-study by the Institutional Review Board of the Medical Faculty of the Goethe-University Frankfurt (decision number: 67/12, file number 122/12). Furthermore the protocol is in accordance to the Clinical Trial Registration Nr. NCT02444871.

Best regards

S. Harder

Prof. Dr. Sebastian Harder
Vorsitzender der Ethikkommission
des Fachbereiches Medizin
der Goethe Universität Frankfurt
